# Clinical investigation on *Theileria equi* and *Babesia caballi* infections in Italian donkeys

**DOI:** 10.1186/s12917-015-0411-z

**Published:** 2015-04-28

**Authors:** Fulvio Laus, Andrea Spaterna, Vanessa Faillace, Fabrizia Veronesi, Silvia Ravagnan, Francesca Beribé, Matteo Cerquetella, Marina Meligrana, Beniamino Tesei

**Affiliations:** Scuola di Bioscienze e Medicina Veterinaria, Università di Camerino, Via Circonvallazione 93/95, 62024 Matelica, (MC) Italy; Dipartimento di Medicina Veterinaria, Università di Perugia, 06100 Perugia, Italy; Istituto Zooprofilattico Sperimentale delle Venezie, 35020 Legnaro, (PD) Italy

**Keywords:** Donkey, Piroplasmosis, Tick borne diseases, *Theileria equi*, *Babesia caballi*

## Abstract

**Background:**

Interest in the welfare and diseases of donkeys is constantly increasing in several countries. Despite this, clinical research into donkeys needs to be in continual development since they show different reactions compared to horses in many conditions, including infectious diseases, and need specific clinical and therapeutic approaches. No reports are currently available on clinical and clinical pathology data regarding donkeys with natural piroplasms infection.

**Results:**

Venous blood samples were taken from one hundred and thirty eight donkeys and underwent indirect fluorescent antibody test (IFAT) to detect IgG antibodies against *Theileria equi* and *Babesia caballi* and real-time polimerase chain reaction (PCR) to detect *Babesia* spp. and *Theileria* spp. Clinical examinations, haematological analyses and serum bilirubin evaluation were also performed and compared with positive or negative status. A seroprevalence of 40.6% and 47.8% was found for *T. equi* and *B. caballi*, respectively; double positivity was detected in 19.6% of the animals. PCR results showed that 17.4% of the animals tested positive for *T.equi* and 3.6% for *B. caballi* with no double positivity. Twelve donkeys (8.7%) had clinical signs consistent with chronic forms of the disease and no acute forms were detected. Fifty-eight donkeys had haematological and serum bilirubin alterations and 56 (96.6%) of them were IFAT and/or PCR positive. Changes in erythrocyte number, packed cell volume, hemoglobin concentration, mean corpuscular hemoglobin, platelets number and total bilirubin were significantly associated with positive and symptomatic animals.

**Conclusion:**

Nonspecific clinical presentation seems to be very common in donkeys and several clinical pathology alterations persist after natural infection. Therefore, apparently healthy donkeys can have masked but severe clinical pathology alterations. Acute forms are very seldom observed in donkeys. Clinical monitoring of chronically infected donkeys is recommended since such animals represent a risk both for transmission to other animals and for their own health; furthermore, their production performances could be reduced. The study should also be intended as a contribution for veterinary practitioners because it describes the most usual clinical presentations and laboratory findings of equine piroplasmosis in naturally infected donkeys in endemic areas.

## Background

Interest in the welfare and diseases of donkeys is constantly increasing in several countries, mostly due to the recent rediscovery of donkey milk as an alternative food source for milk-intolerant children. Clinical research on donkeys needs to be in continual development since they have different reactions compared to horses in many conditions (e.g. resistance to pain in case of colic), including infectious diseases, making it more difficult to recognize the symptoms normally observed in horses [1,2]. Their current popularity is also due to their use as pets, in addition to recreational purposes, sports activities, donkey-assisted therapy and, to a lesser extent, as pack/draught animals and for meat production [3].

Equine piroplasmosis (EP) is a tickborne disease caused by the protozoa *Babesia caballi* and *Theileria equi*. EP is endemic in most equine populations in tropical and subtropical areas of the world and affects all equid species, including horses, donkeys, mules, and zebras [4,5]. Chronic cases are more common in donkeys than horses and are usually characterized by nonspecific clinical signs such as lethargy, partial anorexia, poor work performance and body weight loss [6]. In acute forms of EP, donkeys can show fever, listlessness, depression, noticeable thirst, swelling of the eyelids, constipation, presence of yellow mucous covering feces, yellowish coloration of urine, and splenomegaly [6]. Donkeys can also show an asymptomatic form of the infection and, in comparison to horses, they also show a lower parasitemia [6]. The most common hematological alteration found in horses is decreased packed cell volume, hemoglobin and erythrocyte number, in addition to hyperbilirubinemia and thrombocytopenia [3]. After subclinical, chronic or acute infection, donkeys usually remain asymptomatic carriers with positive antibody titers throughout life [6].

Several data are available for the epidemiology of equine piroplasmosis (EP) in horses [7,8] and information on its epidemiology in Italy has also been reported [9-13]. However, few reports exist regarding the prevalence of these parasites in donkeys [14-17] and, to the authors’ knowledge, only few surveys have been carried out in Italy [18-20]. The present study is the first investigation aimed at evaluating and comparing the direct (by PCR) and indirect (by IFAT) presence of *B. caballi* and *T. equi* with clinical signs and clinical pathology data in naturally infected donkeys in Italy.

## Methods

One hundred and thirty eight mixed breed donkeys (109 females, 7 stallions and 22 geldings) ranging from 1 to 22 years of age (mean 7.6, d.s. = 4.7) belonging to 8 different farms (mean herd size 17 donkeys, d.s. 6 donkeys) in central Italy were included in the study. The area was chosen due to the high prevalence of tickborne pathogens previously found in equids [12,13,18,21,22], the proven presence of the tick vectors [23] and because vet practitioners have frequently reported heavy tick infestations in equids. All the animals were born and reared in Italy and had never been moved out of the country. The survey was performed between March and October 2013 in farms of varying nature and size, including herds for milk production (n = 5), onotherapy centers (n = 2) and private facilities (n = 1) where animals were reared for leisure. De-worming and topical ectoparasite repellents were regularly administered to all the animals who were free from ticks at the moment of evaluation.

A general clinical examination was performed on each donkey; the evaluation also included a body condition score (BCS) estimation, following the scheme of Pearson and Quassat (1996) [24]. Donkeys showing clinical signs not attributable to EP (e.g. lameness) were excluded from the study to avoid interference on blood analysis. Venous blood samples were collected from each donkey from the jugular vein into sterile tubes with (two tubes) and without (one tube) ethylenediaminetetraacetic acid (EDTA) and maintained at +4°C. The samples with EDTA were submitted for a complete blood count (CBC), which included: erythrocytes count (RGB), packed cell volume (PCV), hemoglobin (Hb), mean corpuscular volume (MCV), mean corpuscular hemoglobin (MCH), mean corpuscular hemoglobin concentration (MCHC), total leukocytes, neutrophils, lymphocytes, monocytes, eosinophils, basophils and platelets (Cell Dyn 3500, Abbott). Moreover, an aliquot of 200 μl was destined to genomic DNA extraction using the QIAamp DNA Blood Mini kit (QIAGEN S.p.A., Milan, Italy) according to the manufacturer’s instructions. To ensure the effectiveness of the nucleic acid extraction, a PCR targeting the 18S rRNA was applied [25]. The extracted DNA was submitted to a Real Time PCR Sybr Green assay to detect 509 base pairs of 18S rRNA gene of *Babesia* spp. and *Theileria* spp. using the primer BJ1 and BN2 described by Casati *et al.* (2006) [26]. The method shows a sensitivity of 10^3 DNA copies/μl. The species identity was determined by subsequent amplicon sequencing. All PCR products were sequenced using the Big Dye Terminator v 3.1 cycle sequencing kit (Applied Biosystem, Foster City, CA, USA) in a 16-capillary ABI PRISM 3130 × l Genetic Analyzer (Applied Biosystem, Foster City, CA, USA). Sequence data were assembled and edited with SeqScape software v 2.5 (Applied Biosystem, Foster City, CA, USA), aligned and compared with representative sequences available in GenBank [27].

Samples without EDTA were centrifuged at 4000 rpm for 10 minutes; the separated sera were collected and divided into two aliquots. The first aliquot was used for dosage of total bilirubin (TB) (Targa 3000 plus, Biotecnica Instruments); the second was utilized to determine the presence of IgG antibodies against *T. equi* and *B. caballi* using a commercial indirect fluorescent antibody test (IFAT) (MegaScreen®, 112 DIAGNOSTIK MEGACORE Laboratories, Horbranz, Austria).

### Statistical analysis

Prevalence and 95% binomial confidence intervals (CI) were calculated [28] for the serologic and molecular test results.

Hematological parameters and serum bilirubin were tested for normality by Kolmogorof-Smirnov test and then analyzed by ANOVA or Mann–Whitney *U*-test for comparison between positive (both to PCR and/or IFAT) and negative animals and between donkeys with and without clinical signs. Comparison of the clinical pathology results with normal reference values [29] was carried out using a t-student’s test. The Chi-square test was performed to evaluate the differences between IFAT and PCR prevalence for *B. caballi* and *T. equi*.. Statistical significance was assessed at the 0.05 probability level in all analyses.

All statistical analyses were performed using the WINPEPI (PEPI-for-Windows) computer program (Epidemiol. Perspect. Innov. 1:6. Available from: http://www.biomedcentral.com/1742-5573/1/6).

### Ethical statement

Tha autor state that the work has been carried out in compliance with relevant guidelines regarding ethical use of animals, approved by the Universitary Ethical Commettee for Animal Protection and in adherence to a high standard (best practice) of veterinary care.

## Results

Ninety-five (68.8%) and 29 (21.0%) donkeys tested positive by IFAT and PCR respectively. The results of the serological and molecular tests performed on blood samples are reported in Table 1, as well as the prevalence (%) and 95% confidence interval (CI) of single and mixed-infections.

**Table 1:** **Number of donkeys, prevalence and confidence interval of the equine tick-borne infections investigated using serological and molecular testing**

	**IFAT**		**PCR**	
**Pathogens**	**No. of positive samples (n = 138)**	**Prevalence (95% CI)**	**No. of positive samples (n = 138)**	**Prevalence (95% CI)**
*T. equi*	56	40.6% (32.3-49.3)	24	17.4% (11.5-24.4)
*B. caballi*	66	47.8% (39.3-56.5)	5	3.6% (1.2-8.3)
Single infection				
*T. equi*	29	21.0% (14.6-28.8)	24	17.4% (10.3-23.1)
*B. caballi*	39	28.3% (20.9-36.6)	5	3.6% (0.5-6.2)
Double infection				
*T. equi + B. caballi*	27	19.6% (13.3-27.2)	0	0

IFAT, indirect fluorescent antibody test; PCR, Polymerase Chain Reaction; CI, confidence interval.

All herds (100%) resulted positive at IFAT for both *T. equi* and *B. caballi* and at PCR for *T. equi*. Three herds (37.5%) resulted positive for *B. caballi* at PCR. The prevalence rates within herd are reported in Table 2.

**Table 2:** **Intra-herd prevalence of** ***Babesia caballi*** **and** ***Theileria equi***

**Herd**	**Number of donkeys**	**IFAT prevalence** ***B. caballi***	**PCR prevalence** ***B. caballi***	**IFAT prevalence** ***T. equi***	**PCR prevalence** ***T. equi***
1	24	58,3%	8,3%	45,8%	12,5%
2	15	40,0%	0,0%	20,0%	13,3%
3	8	37,5%	0,0%	25,0%	12,5%
4	17	52,9%	0,0%	47,1%	29,4%
5	14	64,3%	14,3%	50,0%	28,6%
6	19	47,4%	0,0%	42,1%	21,1%
7	27	44,4%	0,0%	40,7%	14,8%
8	14	28,6%	7,1%	42,9%	7,1%

IFAT, indirect fluorescent antibody test; PCR, Polymerase Chain Reaction.

The seroprevalence of *B. caballi* resulted higher than that of *T. equi* but the difference was not statistically significant (P = 0.3). The percentage of PCR positive animals resulted statistically higher for *T. equi* than *B. caballi* (P < 0.001).

Nine (6.5%, 95% CI: 3.0-12.90%) animals were simultaneously IFAT and PCR positive for *T. equi* while 15 (10.9%, 95% CI: 6.2-17.30%) were only PCR positive. None of the IFAT positive donkeys resulted PCR positive for *B. caballi*.

Abnormal clinical pathology data with respect to normal ranges were detected in 58 (42.0%) samples. Fifty-six (96.6%) of these donkeys resulted IFAT and/or PCR positive. Hematological alterations included decreased RGB (n = 49), decreased PCV (n = 24), decreased Hb (n = 31), increased MCH (n = 16), increased MCHC (n = 9) increased WBC (n = 6), increased neutrophils (n = 7), increased eosinophils (n = 5), decreased platelets (n = 20), and increased bilirubin (n = 19). Among IFAT positive donkeys, 46 (48.4%) had one or more hematological and/or bilirubin alteration: 18 (39.1%) proved positive for *B.caballi*, 18 (39.1%) for *T. equi* and 10 (21.7%) were double positives. Among PCR positive donkeys, 19 (65.5%) had one or more hematological and/or bilirubin alteration: 1 (5.3%) was positive for *B. caballi* and 18 (94.7%) were positive for *T. equi*. The statistical differences between positive (both to PCR and/or IFAT) and negative animals are reported in Table 3.

**Table 3:** **Mean, standard deviation and statistical association with negative donkeys of hematobiochemical paramethers**

	**Negative**	**IFAT positive**			**PCR positive**		**PCR and IFAT positive**
**Parameters**	**Mean (sd)**	**B. caballi mean (sd)**	**T. equi mean (sd)**	**Double positives mean (sd)**	**B. caballi mean (sd)**	**T. equi mean (sd)**	**T. equi mean (sd)**
RGB (10^6^/μl)	6.7 (0.8)	5.1 (0.8)	5.0 (0.9)	5.1 (1.0)	5.9 (1.1)	4.7 (0.8)	4.1 (0.4)
		*P* < 0.001	*P* < 0.001	*P* < 0.001	*P* = 0.1	*P* < 0.001	*P* < 0.001
PCV %	35 (5)	32 (4)	32 (5)	31 (7)	33 (2)	29 (3)	31 (6)
		*P* = 0.004	*P* = 0.06	*P* = 0.02	*P* = 0.3	*P* < 0.001	*P* = 0.02
Hb (g/dl)	12.1 (1.3)	10.7 (1.4)	10.5 (2.4)	10.4 (2.2)	10.9 (1.1)	9.1 (1.4)	7.9 (0.9)
		*P* < 0.001	*P* = 0.007	*P* = 0.002	*P* = 0.06	*P* < 0.001	*P* < 0.001
MCV (fl)	52.5 (3.8)	55.1 (3.5)	54.0 (8.0)	56.0 (3.8)	51.3 (4.3)	54.2 (3.1)	55.6 (4.2)
		*P* = 0.7	*P* = 0.6	*P* = 0.3	*P* = 0.5	*P* = 0.5	*P* = 0.3
MCH (pg)	19.1 (1.5)	20.7 (2.0)	20.3 (1.4)	21.0 (2.0)	19.0 (1.7)	23.7 (4.3)	22.3 (1.8)
		*P* = 0.002	*P* = 0.01	*P* < 0.001	*P* = 0.9	*P* < 0.001	*P* < 0.001
MCHC (g/dl)	34.2 (1.5)	35.0 (1.7)	34.9 (1.4)	35.2 (2.2)	35.3 (1.9)	34.9 (1.5)	36.1 (2.0)
		*P* = 0.06	*P* = 0.1	*P* = 0.07	*P* = 0.2	*P* = 0.2	*P* = 0.006
WBC (10^3^/μl)	8.9 (2.9)	9.1 (2.7)	10.1 (2.6)	8.9 (2.4)	9.9 (2.9)	8.7 (2.3)	9.9 (1.7)
		*P* = 0.8	*P* = 0.2	*P* = 0.9	*P* = 0.5	*P* = 0.8	*P* = 0.3
Neutrophils (10^3^/μl)	5.1 (2.4)	4.3 (1.7)	4.4 (0.9)	4.3 (1.9)	5.2 (1.7)	4.8 (1.6)	6.5 (2.9)
		*P* = 0.4	*P* = 0.6	*P* = 0.3)	*P* = 0.3	*P* = 0.3	*P* = 0.007
Lymphocytes (10^3^/μl)	4.6 (1.9)	4.8 (1.9)	5.4 (2.1)	5.2 (1.9)	5.4 (1.7)	5.6 (1.6)	5.9 (0.9)
		*P* = 0.6	*P* = 0.2	*P* = 0.4	*P* = 0.3	*P* = 0.1	*P* = 0.06
Monocytes (10^3^/μl)	0.2 (0.2)	0.2 (0.2)	0.3 (0.2)	0.2 (0.2)	0.0 (0.1)	0.3 (0.2)	0.1 (0.2)
		*P* = 0.6	*P* = 0.05	*P* = 0.3	*P* = 0.1	*P* = 0.1	*P* = 0.9
Eosinophils (10^3^/μl)	0.4 (0.3)	0.5 (0.4)	0.4 (0.3)	0.6 (0.4)	0.5 (0.3)	0.6 (0.4)	0.6 (0.5)
		*P* = 0.3	*P* = 0.4	*P* = 0.09	*P* = 0.1	*P* = 0.1	*P* = 0.07
Basophils (10^3^/μl)	0.1 (0.1)	0.0 (0.1)	0.1 (0.1)	0.1 (0.0)	0.1 (0.0)	0.1 (0.1)	0.0 (0.1)
		*P* = 0.3	*P* = 0.7	*P* = 0.3	*P* = 0.07	*P* = 0.9	*P* = 0.6
Platelets (10^3^/μl)	326 (65)	277 (102)	267 (83)	209 (77)	229 (67)	254 (63)	271 (79)
		*P* = 0.04	*P* = 0.01	*P* < 0.001	*P* = 0.006	*P* = 0.002	*P* = 0.05
TB (mg/dl)	0.3 (0.1)	0.3 (0.14)	0.1 (0.2)	0.3 (0.2)	0.2 (0.0)	0.5 (0.3)	0.3 (0.1)
		*P* = 0.3	*P* = 0.6	*P* = 0.3	*P* = 0.2	*P* = 0.005	*P* = 0.3

IFAT, indirect fluorescent antibody test; PCR, Polymerase Chain Reaction; RGB, erythrocytes; PCV, packed cell volume; Hb, hemoglobin; MCV, mean corpuscular volume; MCH, mean corpuscular hemoglobin, MCHC, mean corpuscular hemoglobin concentration; WBC, white blood cell; PLT, platelet; TB, total bilirubin.

Twelve (10.4%) of the positive donkeys presented signs related to chronic piroplasm infection at the moment of evaluation and all of them were positive for at least one test (Table 4). Detected clinical signs included mild depression (n = 11), body condition score ≤ 2 (n = 10), inappetence (n = 10), pale mucous membranes (n = 4) and mild icterus (n = 6). MCH, MCHC, and TB were statistically higher in symptomatic than in negative donkeys while RGB, PCV, Hb, and platelets were lower. When the blood parameters of symptomatic donkeys were compared to asymptomatic/positives, Hb, MCH and TB resulted in being the only statistically different parameters (P = 0.001, P = 0.0005 and P = 0.005, respectively).

**Table 4:** **IFAT/PCR positivity and changed hematology and serum bilirubin in symptomatic donkeys**

**Donkeys**	**Positivity**	**Clinical signs**	**Clinical pathology**
1	Double IFAT	Depression	↓RGB, ↓Hb, ↓PCV, ↑MCH, ↑MCHC, ↑Neutrophils, ↑Eosinophils
		BCS <2	
		Inappetence	
2	Double IFAT	Depression	↓RGB, ↓Hb, ↓PCV, ↑MCH, ↑MCHC
		BCS <2	
		Inappetence	
		Pale MM	
3	Double IFAT	Depression	↓RGB, ↓Hb, ↓PCV, ↓PLT
		BCS <2	
		Inappetence	
4	Double IFAT	Depression	↓RGB, ↓Hb, ↓PCV, ↑Neutrophils, ↓PLT, ↑TB
		BCS <2	
		Icterus	
		Inappetence	
5	Double IFAT	Depression	↓RGB, ↓Hb, ↓PCV, ↓PLT, ↑TB
		BCS <2	
		Icterus	
		Inappetence	
6	Double IFAT	Depression	↓RGB, ↓Hb, ↓PCV, ↑Neutrophils, ↓PLT, ↑TB
		BCS <2	
		Icterus	
		Inappetence	
7	Double IFAT	Depression	↓RGB, ↓Hb, ↓PLT, ↑TB
		BCS <2	
		Inappetence	
8	PCR *T.equi*	Depression	↓RGB, ↓Hb, ↓PCV, ↑MCH, ↑MCHC, ↑TB
		BCS <2	
		Inappetence	
9	PCR *T.equi*	Depression	↓RGB, ↓Hb, ↓PCV, ↓PLT, ↑TB
		Pale MM	
10	PCR and IFAT (*T. equi*)	Depression	↓RGB, ↓Hb, ↓PCV, ↑MCH, ↑MCHC, ↑WBC, ↓PLT, ↑TB
		BCS <2	
		Pale MM	
		Icterus	
		Inappetence	
11	PCR and IFAT (*T. equi*)	Depression	↓RGB, ↓Hb, ↓PCV, ↑MCH, ↑MCHC, ↓PLT, ↑TB
		BCS <2	
		Icterus	
		Inappetence	
12	PCR and IFAT (*T. equi*)	Pale MM	↓RGB, ↓Hb, ↑TB
		Icterus	

IFAT, indirect fluorescent antibody test; PCR, Polymerase Chain Reaction; RGB, erythrocytes; Hb, hemoglobin; PCV, packed cell volume; WBC, white blood cell; PLT, platelet; TB, total bilirubin; MCH, mean corpuscular hemoglobin, MCHC,mean corpuscular hemoglobin concentration, MM mucous membranes.

↑, higher than normal; ↓, lower than normal.

## Discussion

Although not statistically significant, the seroprevalence of *B. caballi* (47.8%) was higher than that of *T. equi* (40.6%) in accordance with the results obtained in donkeys in Italy [18-20] or in other countries [14,16,17]. The percentage of PCR positive animals resulted statistically higher for *T. equi* (17.4%) than *B. caballi* (3.6%) proving that also in donkeys *T. equi* can persist in a subclinical form for longer than *B. caballi* [30].

In general, the chronic and subclinical natural infection in donkeys included in this study seems to be associated with decreased RGB, PCV, Hb and PLT and with increased MCH. A systematic comparison with previous studies carried out on donkeys is not reliable since they are experimental trials aimed at investigating pathogenic mechanisms or the efficacy of drugs or vaccines often on few splenectomised donkeys [31-36].

Almost all the donkeys having one or more hematological disorder resulted IFAT positive for *B. caballi* and/or *T. equi*; the only two negative animals had increased WBC, neutrophils or eosinophils but similar alterations are not usually related to piroplasm chronic infections [37]. These findings are of remarkable importance since these animals (representing almost half of the IFAT positive donkeys), could not have cleared the parasites from their blood after natural infection, but could only have reduced the level beyond the sensitivity of the PCR test [16]. This consideration is also supported by the fact that 8 (66.7%) of the 12 symptomatic donkeys were IFAT but not PCR positive. Most of the alterations were related to hematological signs of anaemia, thrombocytopenia and hemolysis, could suggest the presence of a direct and immune-mediated pathogenic activity of the parasites.. As a consequence of this kind of subclinical infection, the donkeys could have a reduction of their work or production performance, although further specific investigation are needed to verify such occurrence. A similar situation can also occur in horses, in which slight anemia caused by chronic infection can result in poor athletic performances [3]. However, because of the naturally more quiet behavior of donkeys, their resistance to diseases and the more rural type of farming, in this species it could be difficult to recognize some subtle nonspecific alteration (e.g. reduced milk production, slight decrease in work activity) without a careful evaluation. Piroplasmosis should be considered a differential diagnosis in these animals, which should therefore be monitored for risk of stress (e.g. heavy work load, separation from the foal for the lactating jenny). Furthermore, these animals should also be considered potential asymptomatic carriers.

*T. equi* infected donkeys (e.g. PCR positives) showed a higher likelihood of having hematological alterations compared with *B. caballi* infected animals. The only alteration found in donkeys proving positive for *B. caballi* was a decrease in PLT in one subject. This is in contrast with findings reported for horses, where anemia, thrombocytopenia and leukopenia are reported to have a high incidence also in *B. caballi* positive subjects [38]: the low number of positive animals (n = 5) in the present and in the cited paper could have influenced such statistical results. However, none of the *B. caballi* infected donkeys showed clinical symptoms and it is possible to speculate that *T. equi* has a higher pathogenicity than *B. caballi* in donkeys as suggested for horses [16]*.*

*T. equi* infected donkeys also have a higher TB serum level compared both to negative and to other positive donkeys. The recent infection causing hemolytic anemia could be the reason for this condition since donkeys simultaneously PCR and IFAT positive which are supposed to have a less recent infection since they have already developed antibodies, showed no such association.

Neutrophilia was found to be related only to PCR/IFAT positive to *T. equi* (P = 0.006). Acute experimental infection in donkeys can be characterized by a high absolute neutrophil count [6] but, although these donkeys resulted to be infected, none of them showed signs of the acute form of the disease. More investigations are needed to confirm if similar alterations of white blood cells relate to natural piroplasm infection or are due to concomitant subclinical diseases as we suppose.

In the present study, only Hb, MCHC and TB resulted to be statistically different between symptomatic and non-symptomatic animals. It could be possible to speculate that donkeys can control the clinical symptoms after natural infection in endemic areas, but, at the same time, they can have some residual hematochemical alterations similar to those of symptomatic animals, exposing them to the risk of disease or poor performance.

It should also be highlighted that 19 of the 24 donkeys who resulted PCR positive for *T. equi* were found to be free from clinical signs. These animals confirm that subclinical forms are widespread among donkeys reared in endemic areas, as observed in horses [13] Such findings also support the existence of lifelong carriers, which are persistently infected subjects, potentially capable of enhancing the spread of these pathogens.

## Conclusions

The high prevalence of piroplasms and associated hematochemical alterations in non-symptomatic donkeys or in donkeys with minimal clinical evidence found in this study could be more usual than previously considered, especially in areas where piroplasmosis is endemic. Such animals should be monitored for red cells, red cell related parameters and thrombocytopenia because unapparent carriers can occasionally exhibit relapses of the clinical disease associated with stress, strenuous exercise, immunosuppression, and steroid administration. Furthermore, these animals can act as a source of piroplasms for ticks, increasing the probability of transmission to other animals, including horses. Since the effects of this unapparent infection and its possible consequences on the production performance of the donkeys (milk quantity and quality, weight gain, work performance) are not yet known, further studies to investigate such relationships should be performed. Currently there is no suitable pharmacotherapy available to clear the *T. equi* infection from affected donkeys [6]. It has therefore become urgent to act with surveillance plans and preventive therapy. The study should also be intended as a contribution for veterinary practitioners because it describes the most usual clinical presentations and laboratory findings of EP in naturally infected donkeys in Italian endemic areas.

## Abbreviations

EP: Equine piroplasmosis
IFAT: Indirect fluorescent antibody test
PCR: Polymerase chain reaction
CBC: Complete blood count
RGB: Erythrocytes
PCV: Packed cell volume
Hb: Hemoglobin
MCV: Mean corpuscular volume
MCH: Mean corpuscular hemoglobin]
MCHC: Mean corpuscular hemoglobin concentration
WBC: White blood cell
PLT: Platelet
TB: Total bilirubin
EDTA: Ethylenediaminetetraacetic acid
